# Vascular islands during microvascular regression and regrowth in adult networks

**DOI:** 10.3389/fphys.2013.00108

**Published:** 2013-05-16

**Authors:** Molly R. Kelly-Goss, Richard S. Sweat, Mohammad S. Azimi, Walter L. Murfee

**Affiliations:** Department of Biomedical Engineering, Tulane UniversityNew Orleans, LA, USA

**Keywords:** angiogenesis, microcirculation, mesentery, proliferation, endothelial cell, disconnected segment, vascular island

## Abstract

**Objective**: Angiogenesis is the growth of new vessels from pre-existing vessels and commonly associated with two modes: capillary sprouting and capillary splitting. Previous work by our laboratory suggests vascular island incorporation might be another endothelial cell dynamic involved in microvascular remodeling. Vascular islands are defined as endothelial cell segments disconnected from nearby networks, but their origin remains unclear. The objective of this study was to determine whether vascular islands associated with microvascular regression are involved in network regrowth.

**Methods:** Mesenteric tissues were harvested from adult male Wistar rats according to the experimental groups: unstimulated, post stimulation (10 and 70 days), and 70 days post stimulation + restimulation (3 and 10 days). Stimulation was induced by mast cell degranulation via intraperitoneal injections of compound 48/80. Tissues were immunolabeled for PECAM (endothelial cells), neuron-glial antigen 2 (NG2) (pericytes), collagen IV (basement membrane), and BrdU (proliferation).

**Results:** Percent vascular area per tissue area and length density increased by day 10 post stimulation compared to the unstimulated group. At day 70, vascular area and length density were then decreased, indicating vascular regression compared to the day 10 levels. The number of vascular islands at day 10 post stimulation was dramatically reduced compared to the unstimulated group. During regression at day 70, the number of islands increased. The disconnected endothelial cells were commonly bridged to surrounding networks by collagen IV labeling. NG2-positive pericytes were observed both along the islands and the collagen IV tracks. At 3 days post restimulation, vascular islands contained BrdU-positive cells. By day 10 post restimulation, when vascular area and length density were again increased, and the number of vascular islands was dramatically reduced.

**Conclusion:** The results suggest that vascular islands originating during microvascular regression are capable of undergoing proliferation and incorporation into nearby networks during network regrowth.

## Introduction

The design of therapies aimed at manipulating the microcirculation requires a greater understanding of the cellular dynamics involved in angiogenesis in adult tissues. Angiogenesis is defined as the growth of new vessels from existing vessels and is most commonly described by capillary sprouting and capillary splitting (Carmeliet and Tessier-Lavigne, 2005). Capillary sprouting involves the proliferation and migration of endothelial cells from an existing vessel. Whereas capillary splitting, termed intussusception, involves the formation of intra-luminal pillars within a single vessel via endothelial cell filopodia and the eventual formation of two separate lumens.

Recently, work by our laboratory suggested that vascular island incorporation is a potential third endothelial cell dynamic involved in angiogenesis (Kelly-Goss et al., 2012; Stapor et al., 2013). Vascular islands are defined as endothelial cell segments disconnected from a nearby network. The study by Kelly-Goss et al. identified that they are multi-cellular, containing endothelial cells and pericytes, and undergo proliferation and increased branching during angiogenesis. Stapor et al. demonstrated that vascular islands are able to connect to their nearby network during angiogenesis. While these findings support a role for vascular islands in angiogenesis, their origin remains unclear. Mancuso et al. demonstrated that molecular and cellular players left behind after vessel regression provided spatial tracks for rapid regrowth of the vasculature (Mancuso et al., 2006). Collagen IV basement membrane sleeves associated with pericytes remained after Vascular Endothelial Growth Factor Receptor-2 (VEGFR-2) inhibition of tumor growth. Upon removal of an antibody inhibitor, the tumor microvasculature rapidly regrew along the pre-existing tracks (Mancuso et al., 2006). Based on these results, we hypothesized that the endothelial cells left behind during regression are a source for vascular islands and that these cells are also capable of being reused during regrowth of a microvascular network.

The objective of this study was to determine whether vascular islands associated with microvascular regression are involved in network regrowth. Using the rat mesentery, we show that the number of vascular islands, indeed, increases during network regression in an adult tissue. After network restimulation, vascular islands undergo proliferation and decrease in number. Both these findings are consistent with our previous characterization of vascular island dynamics involved in the initial stimulation of the networks (Kelly-Goss et al., 2012). The rat mesentery was selected for our current study to be consistent with this previous study identifying vascular islands and because it allows for observation of intact microvascular networks down to the single cell level. This capability has proven valuable for identifying pericyte and endothelial dynamics during angiogenesis stimulated by tissue exteriorization, mast cell degranulation, and hypoxia to better understand the cellular dynamics involved in angiogenesis in other tissue remodeling scenarios (Ponce and Price, 2003; Murfee et al., 2006; Anderson et al., 2008; Stapor and Murfee, 2012; Sweat et al., 2012). Our current study adds new information regarding the origin for the disconnected endothelial cells that exist as vascular islands. Our results suggest that vascular islands are associated with microvascular regression and that vascular islands can be involved in network regrowth.

## Methods

### Mast cell degranulation model of angiogenesis

**Figure 1:**
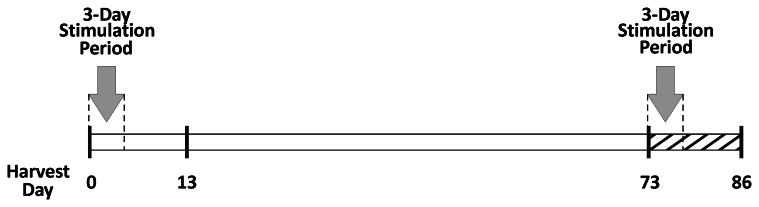
**Experimental timeline.** For the unstimulated group, tissues were harvested on the equivalent of day 0. Initial stimulation involved 3 days of 48/80 injections. On day 13 from the start of the experiment, tissues were harvested for the 10 days post stimulation group. On day 73, tissues were either harvested for the day 70 post stimulation group or re-stimulated with a repeat of the 3 day 48/80 injection protocol. On day 86, tissues were harvested for the 70 days + 10 day re-stimulation group.

All experiments were performed under the guidelines and in accordance with Tulane University's Animal Care and Use Committee. Adult male Wistar rats (350–450 grams; Charles River Laboratories, Wilmington, MA) were treated with a protocol previously established by our laboratory (Murfee et al., 2006; Kelly-Goss et al., 2012; Stapor and Murfee, 2012). Individual 2.5 mL doses of compound 48/80 (Sigma-Aldrich, St. Louis, MO) were administered via intraperitoneal injections twice a day for 3 days in increasing concentrations (140, 280, 420, 560, and 700 μg/mL in sterile saline). Tissues were harvested from the following experimental groups (1) unstimulated (*n* = 4 rats), (2) 10 days post stimulation (*n* = 4 rats), (3) 70 days post stimulation (*n* = 4 rats), and (4) 70 days post stimulation + 10 days restimulation (*n* = 4 rats). A timeline for the experimental protocol is displayed in Figure 1. For restimulation groups, animals received a repeat of the 48/80 injection protocol starting on day 70. Following the repeated 3 day injection protocol, tissues were harvested after 10 days. Compound 48/80 is a mast cell degranulator compound that has been previously shown to stimulate a robust multi-factorial angiogenic response in the adult rat mesenteric networks over a relatively short time period (Norrby et al., 1990; Ponce and Price, 2003; Anderson et al., 2008; Kelly-Goss et al., 2012; Stapor and Murfee, 2012). The rat mesentery and the compound 48/80 stimulation model were selected for this study to be consistent with previous work that identified the presence of vascular islands during angiogenesis (Kelly-Goss et al., 2012). This preceding study demonstrated that endothelial cells along vascular islands undergo proliferation at day 3 post the initial 48/80 stimulation. In the current study, additional tissues were harvested from 2 rats at day 3 post restimulation to qualitatively determine whether cells along vascular islands associated with vascular regression were also capable of undergoing proliferation.

### Tissue harvesting

Rats were anesthetized by an intramuscular injection of ketamine (80 mg/kg bw) and xylazine (8 mg/kg bw). After shaving and sterilizing each rat's abdomen, a single incision between the sternum and pelvic bone was made down the linea alba. The rats were euthanized via 0.15 mL intra-cardiac injection of Beuthanasia (Shering-Plough Animal Health Corp. Union, Kenilworth, NJ). For the 70 days stimulation + 3 days restimulation group, peritoneal cavities were injected with bromodeoxyuridine (BrdU) (1 mg/ml; 30 ml) and left for 2 h. After the incubation time, the rats were euthanized via the same method as the other groups. The first 8–10 vascularized mesenteric windows per rat were dissected starting from the ileum and placed directly in 10 mM Phosphate Buffered Saline (PBS). Mesenteric windows were defined as the thin, translucent connective tissues between the mesenteric arterial/venous vessels feeding the small intestine. The tissues were whole mounted on positively charged slides. Tissues were then fixed in 100% methanol for 30 min at −20°C. Rat mesenteric tissue windows are approximately 20–40 μm thick (Barber et al., 1987) enabling the whole-mounting and visualization of intact networks across their hierarchy. This characteristic of mesentery windows is necessary for the identification of vascular islands.

### Immunofluorescence

After methanol fixation, tissues were labeled for PECAM (endothelial cell marker), neuron-glial antigen 2 (NG2) (pericyte marker), collagen IV (basement membrane marker), or BrdU (proliferating nuclei marker). Tissues underwent one of the following immunolabeling protocols:

*PECAM* + *NG2* + *Collagen IV*: (1) 1:100 rabbit polyclonal NG2 (Millipore, Billerica, MA), 1:200 mouse monoclonal anti-CD31 (PECAM) (BD Pharmingen, San Diego, CA), 1:50 goat polyclonal anti-type IV collagen (Millipore, Billerica, MA), and 1:20 normal donkey serum (NDS) (Jackson ImmunoResearch Laboratories, West Grove, PA) in antibody buffer solution (ABS) (PBS + 0.1% saponin + 2% BSA) at room temperature for 1 h; (2) 1:500 CY3-conjugated streptavidin secondary (Strep-CY3) (Jackson ImmunoResearch Laboratories), 1:50 DyLight-405 AffiniPure donkey anti-goat IgG (H + L) (Jackson ImmunoResearch Laboratories), and 1:20 NDS in ABS at room temperature for 1 h; (3) 1:20 normal goat serum (NGS) in ABS blocking step for 1 h at room temperature; (4) 1:100 Alexa Fluor-488 goat anti-rabbit IgG (GAR-488) (Jackson ImmunoResearch Laboratories) and 1:20 in ABS for 1 h at room temperature.

*PECAM* + *Collagen IV*: (1) 1:200 PECAM, 1:50 collagen IV, and 1:20 NDS in ABS for 1 h at room temperature; (2) 1:500 strep-CY3, 1:50 Alexa Fluor-488 AffiniPure donkey anti-goat IgG (H + L) (DAG-488) (Jackson ImmunoResearch Laboratories) and 1:20 NDS in ABS for 1 h at room temperature.

*PECAM* + *NG2*: (1) 1:200 PECAM, 1:100 NG2, and 1:20 NGS in ABS for 1 h at room temperature; (2)1:500 strep-CY3, 1:100 GAR-488, and 1:20 NGS in ABS for 1 h at room temperature.

*PECAM* + *BrdU*: (1) 6 N HCl at 37°C for 1 h; (2) 1:100 monoclonal mouse anti-bromodeoxyuridine (BrdU) (Dako, Denmark) with 5% NGS in ABS overnight at 4°C; (3) 1:100 goat anti-mouse CY2 (GAM-CY2, Jackson Immunochemcals Inc., PA) in ABS at room temperature for 1 h; (4) 1:200 PECAM in ABS at room temperature for 1 h; (5) 1:500 strep-CY3 in ABS at room temperature for 1 h.

Following each step, tissues were washed for three ten-minute intervals with PBS + 0.1% saponin. All tissues were sealed and preserved in a 50:50 glycerol:PBS solution.

### Microscopy and image acquisition

Images were digitally captured by the following systems: an inverted microscope (Olympus IX70, Olympus America, Inc., Melville, NY) coupled with a Photometrics CoolSNAP EZ camera using 4× (dry; Numerical Aperture = 0.1), 10 × (dry; *NA* = 0.3), 20 × (oil; *NA* = 0.8 or dry; *NA* = 0.75), and 60× (oil; *NA* = 1.4) objectives. The projection image shown in Figure 4 was obtained with a Ziess LSM 510 META confocal microscope using a Ziess 40×/NA = 1.3 oil objective.

### Quantification of microvascular remodeling and vascular islands

The percent vascular area per tissue area, vascular length density, and the number of vascular islands per vascular length density were quantified per experimental group: (1) unstimulated, (2) 10 days post stimulation, (3) 70 days post stimulation, (4) 70 days post stimulation + 10 days restimulation. From the harvested tissues, 4 vascularized tissues per animal were randomly selected (*n* = 4 rats per group) for analysis. Measurements for each metric were made per tissue. The 4 tissues per animal were then averaged and this average was used as the value per animal. A montage of each tissue was generated by the overlaying of 4× images and used for measurement of tissue and vascular area. Tissue area was defined as the entire area of the mesenteric window. Vascular area was defined as the cumulative area circumscribed around all microvascular networks in the tissue. For vascular density measurements, two 4× fields of view were randomly selected per tissue and the total length of PECAM positive vessels was divided by the corresponding circumscribed vascular area. If a single 4× field of view was avascular, then it was not used and another field of view was again randomly selected. Measurements were made using ImageJ (US National Institutes of Health, Bethesda, MD). The number of vascular islands per vascular area for each tissue was counted under the microscope using a 10× objective. Vascular islands were defined as PECAM-positive endothelial cell segments disconnected from the nearby blood microvascular network. Disconnected segments were confirmed based on discontinuous PECAM labeling by focusing through the thickness of the tissue with the 20× or 60× objective. Previous characterization of vascular islands confirmed that the discontinuous PECAM labeling was associated with a lack of luminal connection with the nearby network (Kelly-Goss et al., 2012). Vascular islands were not included in the vascular area or vascular length measurements. For unstimulated and 70 days post stimulation groups, the percentage of vascular islands containing a NG2 positive pericyte, the percentage of vascular islands bridged by a NG2 positive pericyte to a another blood vessel segment, and the percentage of vascular islands bridged by collagen IV labeling to a another blood vessel segment were quantified in each tissue (*n* = 4 rats per group). Pericytes were identified based on positive NG2 labeling and a characteristic elongated cell morphology.

### Statistical analysis

Percent vascular area per tissue area, vascular length density, and the number of vascular islands per vascular length density metrics were compared across the experimental groups using a One-Way ANOVA on Ranks followed by a Student-Newman-Keuls pairwise comparison test. An ANOVA on Ranks was used for the comparisons because the data was not normally distributed. A Student's *t*-test was used to compare the percentage of vascular islands containing a NG2-positive pericyte, the percentage of vascular islands bridged by a NG2-positive pericyte to a another blood vessel segment, and the percentage of vascular islands bridged by collagen IV labeling to another blood vessel segment between the unstimulated and 70 day post stimulation groups. All statistical comparisons were made using SigmaStat (Systat Software, Inc., Chicago, IL). Statistical significance was accepted for *p* < 0.05. Values are presented as means ± SEM.

In order to evaluate inter-animal variances the averages of the tissues analyzed for each metric (percent vascular area per tissue area, vascular length per vascular area, and the number of islands per vascular length density) were compared across rats within the unstimulated and day 10 experimental groups using a One-Way ANOVA. No significance difference across rats within each experimental group was found suggesting that metrics were not animal dependent. Also, using a Mann–Whitney *U*-test, the averages for each metric were compared across the current study and our previous study (Kelly-Goss et al., 2012) for both unstimulated and day 10 groups. The averages were also not significantly different.

## Results

**Figure 2:**
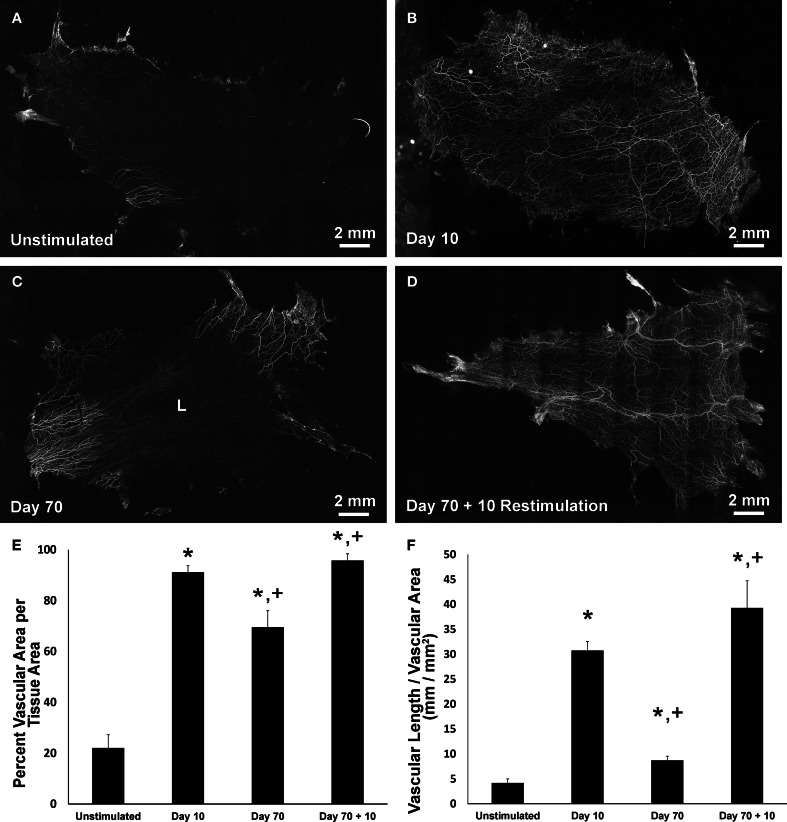
**Microvascular network area and length density changes post stimulation and restimulation. (A–D)** Representative montages of PECAM-labeled mesenteric tissues from the following experimental groups: unstimulated, 10 day post stimulation, 70 day post stimulation, and 70 day post stimulation + 10 day restimulation. Lymphatic vasculature is indicated by “L.” **(E)** Quantification of vascular area per tissue area. **(F)** Quantification of vascular length per vascular area. ^*^p < 0.05 compared to the unstimulated group, ^+^*p* < 0.05 compared to the previous time point. Values are means ± SEM.

In all tissues, PECAM labeling identified vessel segments along the hierarchies of microvascular networks (Figures 2A–D). Similar to previous reports (Kelly-Goss et al., 2012; Stapor and Murfee, 2012; Sweat et al., 2012), compound 48/80 stimulation caused a significant increase in vascularized area and vascular length density at day 10 compared to unstimulated tissues (Figures 2E,F). At day 70 post stimulation, microvascular networks displayed significant decreases in area and vessel density compared to day 10 (Figures 2B,C). The decreases in angiogenic metrics indicate that by this time point networks have undergone regression. Restimulation of the day 70 tissues caused microvascular network regrowth (Figures 2C,D) indicated by a significant increase in both vascular area and density (Figures 2E,F).

**Figure 3:**
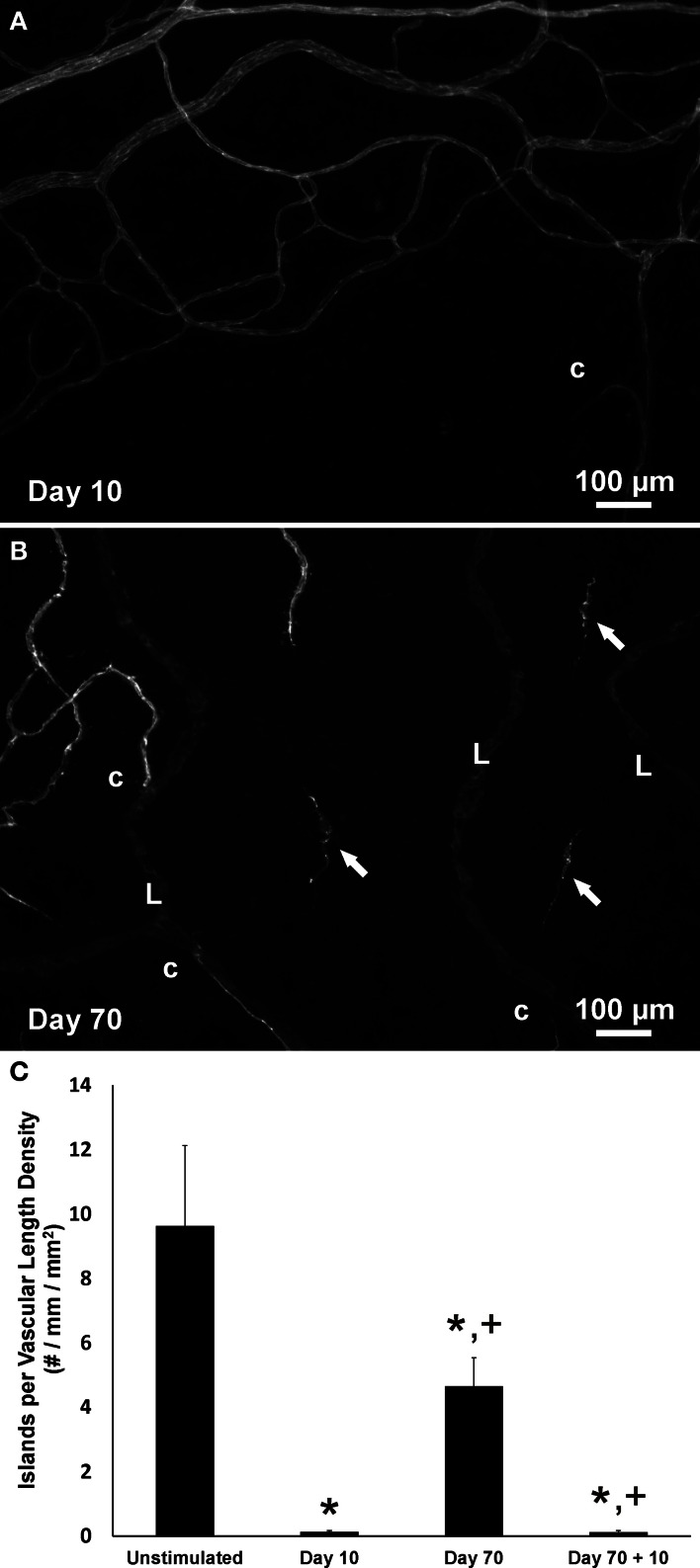
**The presence of vascular islands is associated with network regression. (A,B)** Representative images of the periphery of vascular networks labeled for PECAM at day 10 **(A)** and at day 70 post stimulation **(B)**. At day 70 during microvascular network regression, the number of vascular islands (arrows) is increased. Blind ended capillary segments still connected to a network are indicated by “c.” Lymphatic vessels are indicated by “L.” **(C)** Quantification of the number of vascular islands per vascular length density over the time course of growth, regression, and regrowth. ^*^p < 0.05 compared to the unstimulated group, ^+^*p* < 0.05 compared to the previous time point. Values are means ± SEM.

**Figure 4:**
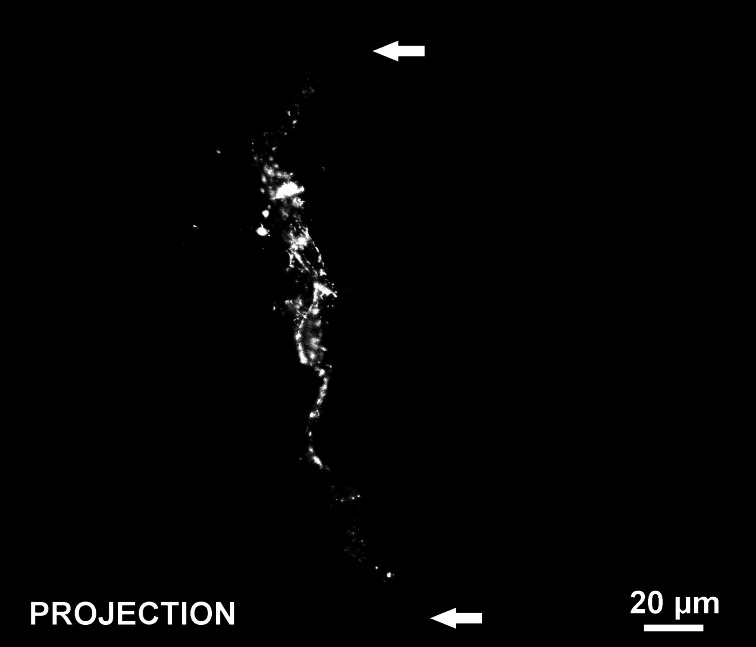
**Confocal projection of optical sections throughout the thickness of a PECAM-positive vascular island at day 70 post stimulation.** The disconnection of the endothelial cell segment from nearby vasculature is confirmed by the voids in PECAM labeling at either end of the vascular island (arrows).

**Figure 5:**
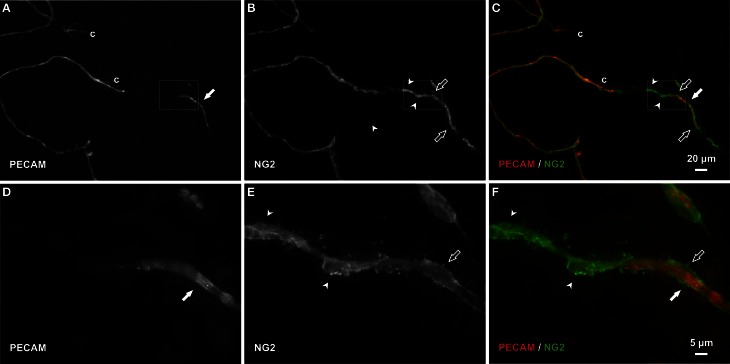
**Pericytes bridge and extend along vascular islands during regression. (A–C)** Examples of PECAM-positive endothelial cells along vascular islands (filled arrows) with positive NG2 pericyte association. NG2 positive pericytes were observed associating with islands by either wrapping along (open arrows) or bridging (arrowheads) endothelial cells. **(D–F)** Higher magnification images of PECAM and NG2 labeling within the area defined by the rectangle. Blind ended capillary segments still connected to a network are indicated by “c.”

**Figure 6:**
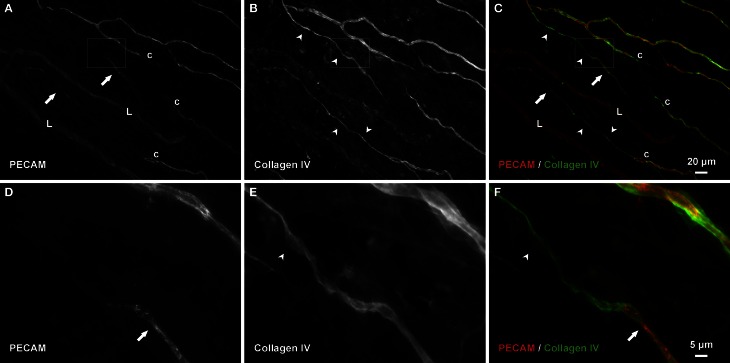
**Collagen IV tracks connect vascular islands to nearby networks and other vascular islands. (A–C)** An example of PECAM-positive endothelial cells along vascular islands (arrows) with positive collagen IV connections to nearby networks (arrowheads). **(D–F)** Higher magnification images of PECAM and collagen IV labeling within the area defined by the rectangle. Blind ended capillary segments still connected to a network are indicated by “c.” Lymphatic vessels are indicated by “L.”

**Figure 7:**
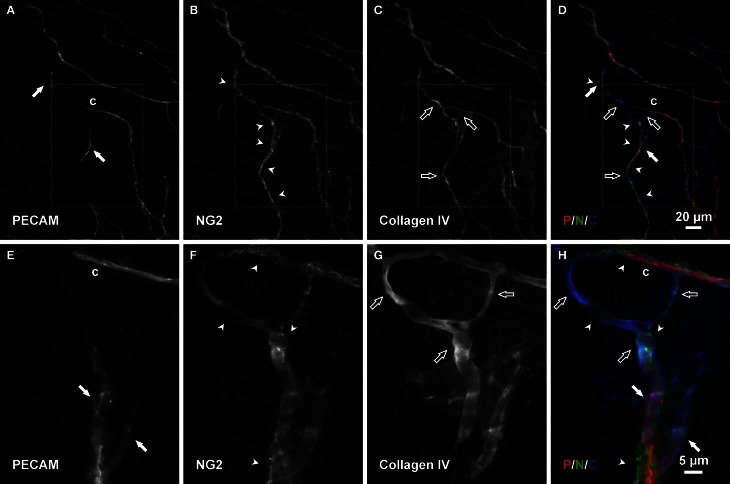
**Connecting basement membrane tracks contain pericytes. (A–D)** Examples of PECAM positive endothelial cells along a vascular island (filled arrows) connected to a nearby vessel segments via an NG2/collagen IV track. Arrowheads indicate pericytes; open arrows indicate basement membrane. **(E–H)** Higher magnification images of PECAM, NG2, and collagen IV labeling within the area defined by the rectangle. Blind ended capillary segments still connected to a network are indicated by “c.”

Consistent with our initial characterization of vascular islands during angiogenesis (Kelly-Goss et al., 2012), the number of vascular islands dramatically decreased at day 10 post stimulation vs. the unstimulated level (Figure 3C). During the state of regression at day 70, the total number of vascular islands per tissue and the normalized number of vascular islands per vascular density increased (Figures 3A–C). This increase suggests that the presence of vascular islands temporally correlates with regression. The majority of vascular islands at day 70 were located at the periphery of a regressing network (Figure 3B). The endothelial cells along vascular islands at day 70 were confirmed to be disconnected from nearby networks using confocal microscopy. A projection of optical sections through a vascular island identifies gaps in PECAM labeling at both ends of the endothelial cell segment (Figure 4). Vascular islands at day 70 were commonly associated with NG2-positive pericyte wrapping (Figure 5). Also, all of the PECAM-positive vascular islands at day 70 displayed collagen IV labeling and most of the islands were bridged to the nearby network via a collagen IV track (Figure 6). Similar to Mancuso et al.'s description of collagen sleeves left behind after VEGF inhibition in tumors (Mancuso et al., 2006), the collagen IV bridges associated with regressing networks in the rat mesentery commonly contained pericytes (Figure 7). The percentages at day 70 were significantly increased compared to unstimulated levels (vascular islands with pericyte wrapping at 58 ± 3% vs. 39 ± 4%; vascular islands with pericyte bridges at 41 ± 4% vs. 10 ± 3%; and vascular islands with collagen IV bridges at 83 ± 2% vs. 42 ± 3%, respectively; *p* < 0.01 for all comparisons). Pericyte and collagen IV metrics were not quantified at day 10 or day 70 + restimulation because for these experimental groups, the number of vascular islands were dramatically decreased and too few for analysis (Figure 3C).

**Figure 8:**
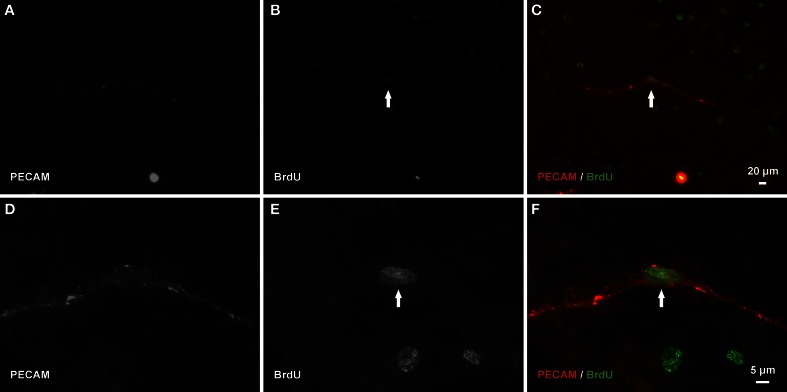
**Endothelial cells along vascular islands undergo proliferation during angiogenesis post restimulation. (A–C)** Example BrdU-positive nucleus (arrow) along a PECAM positive vascular island. **(D–F)** Higher magnification images of PECAM and BrdU labeling of the same vascular island.

After network regrowth, the number of vascular islands again dramatically decreased (Figure 3C). Day 10 post restimulation represents a time point at which a network has just undergone angiogenesis. Evidence for the vascular islands present at day 70 being involved in this regrowth is supported by observation of positive BrdU labeling of endothelial cell nuclei within PECAM positive vascular islands at day 3 post restimulation (Figure 8).

## Discussion

The results of this study suggest that vascular islands, defined as endothelial segments disconnected from a nearby network, originate during microvascular regression. Previous work from our laboratory identified the presence of vascular islands in unstimulated tissues and showed that they are capable of undergoing proliferation and branching (Kelly-Goss et al., 2012) during angiogenesis. In another study in our laboratory, Stapor et al. established the culturing of rat mesenteric tissues as a tool for investigating angiogenesis and demonstrated that vascular islands are capable of physically connecting to nearby networks by comparing images of the same network before and after serum stimulated network growth (Stapor et al., 2013). Our current study adds new information by addressing the origin of the endothelial cells for the vascular islands. Logical cell origins include endothelial cells left behind during vascular regression and the differentiation of new cells from a tissue resident progenitor cell population. Our results implicate vessel regression as a source for the disconnected endothelial cell segments (Figures 2, 3) and further suggest that these vascular islands associated with regression are still capable of undergoing proliferation and incorporation into a network (Figures 3, 8).

The ability for endothelial cells along a vascular island to incorporate with a nearby network during microvascular growth is supported by similar dynamics observed in other remodeling scenarios. For example, during development, vasculogenesis involves the connection of individual endothelial cell chords into a vascular plexus (Risau, 1997; Drake, 2003). During angiogenesis in the adult, circulating endothelial progenitor cells have been suggested to incorporate into existing vessels (Asahara et al., 1997; Tepper et al., 2003). Also, microvessel fragments cultured in three-dimensional collagen gels have been shown to connect with a host vasculature after implantation (Hoying et al., 1996; Nunes et al., 2010). Finally, lymphatic vascular islands have been associated with migration and recruitment of cells during adult lymphangiogenesis (Ohtani and Ohtani, 2001; Rutkowski et al., 2006; Stacker and Achen, 2008).

To our knowledge, our laboratory was the first to show that blood vascular islands, capable of contributing to angiogenesis, exist in an adult tissue. Work by Hughes and Chang-Ling, described the presence of apoptotic isolated endothelial cell segments during vascular regression in the retina (Hughes and Chang-Ling, 2000). Our current results indicate that vascular islands in rat adult mesenteric microvascular networks are also associated with regression, and further suggest that at least a population of these disconnected segments can be recruited to contribute to network growth. While the physiological relevance of vascular islands in the rat mesentery remain unclear, understanding their involvement in angiogenesis might provide therapeutic insight into the source of newly formed vessels after the removal of an anti-angiogenic treatment. In a recent commentary, Kozin et al. focused on the issue of tumor neovascularization after non-curative radiotherapy (Kozin et al., 2012). Questions regarding the source of the new vessels in relapsing tumors and whether or not endothelial cells that survive are able to re-establish a network remain unanswered (Kozin et al., 2012). For now our results indicate that vascular islands formed during network regression are capable of providing a source of endothelial cells for network regrowth in an adult tissue. While future studies will be required to determine whether vascular island incorporation influences network growth rate and/or metabolic efficiency, our results support a novel endothelial cell dynamic that motivates follow-up, more mechanistic investigations.

During rapid regrowth of tumor microvessels after the removal of a VEGFR-2 inhibitor, endothelial cell sprouts have been shown to track along pericyte-lined, collagen IV-positive basement membrane sleeves left behind by vessel regression (Inai et al., 2004; Mancuso et al., 2006). We show similar pericyte-lined collagen IV tracks in regressed rat mesenteric networks post an initial inflammatory stimulus. Our results suggest that endothelial cells might also be left behind during regression and these cells can also be reused by networks. During the initial stages of angiogenesis post network restimulation, endothelial cells along vascular islands are proliferative. By 10 days, a time point after angiogenesis has occurred, the number of vascular islands dramatically decreases suggesting that the vascular islands have reincorporated with the network. Since vascular pericytes have been reported to both stabilize and potentially guide capillary sprouts (Gerhardt and Betsholtz, 2003; Ponce and Price, 2003), we speculate that pericytes stabilize and/or guide vascular islands along the existing collagen IV tracks. In the context of our mast cell degranulation stimulus, histamine has been shown to induce pericyte contraction (Kelley et al., 1988; Murphy and Wagner, 1994), and pericyte contraction has been implicated as a mediator of endothelial cell-matrix interactions critical for angiogenesis (Lee et al., 2010). Assuming pericytes play similar roles on capillary sprouts and vascular islands, pericytes might help facilitate vascular island extension toward the nearby network or vice versa. This potential role for pericytes highlights the need to investigate the molecular mechanisms of the vascular island incorporation process, which likely includes the extension of endothelial cells from both the vascular island and a nearby capillary sprout. Future studies are also needed to identify the temporal dynamics of vascular island lumen closure during regression and the subsequent opening and connection during reincorporation.

Day 70 was selected to capture network regression post compound 48/80 stimulation based on the previous characterization of 48/80 stimulated angiogenesis in adult rat mesentery networks (Jakobsson, 1994; Amos et al., 2008). Both studies indicate that vascular length density decreases post 48/80 stimulation by day 60. Consistent with these results, we show in the current study that at day 70 both density and area have significantly decreased compared to day 10. We also show that vascular islands at day 70 displayed increased pericyte coverage, pericyte bridging and collagen IV bridging to nearby networks compared to vascular islands in unstimulated tissues. We speculate that the majority of vascular islands in unstimulated vs. day 70 tissues are associated with different stages of the regression process. Our previous work (Kelly-Goss et al., 2012) noted that vascular islands in unstimulated networks are not TUNEL-positive. While we cannot rule out the possibility that a percentage of vascular islands at day 70 are apoptotic, our results do indicate that they are able to proliferate. Future studies will be needed to determine whether the presence of existing collagen IV, pericyte-positive tracks influences the rate of reconnection and to identify the unknown dynamics related to vascular regression and the fate of vascular islands when a network is not stimulated to undergo growth.

A limitation of the current study remains that vascular islands were not tracked over time. Hence, individual endothelial cell segments were not shown to directly connect to a nearby network. While the observation of endothelial cell proliferation and the decrease in the number of vascular islands during network regrowth support vascular island reincorporation, definitive evidence is not provided. We do know that (1) vascular islands can connect to networks (Stapor et al., 2013) and (2) that at 10 days post 48/80 stimulation the hierarchy of the remodeled network, including blind-ended capillary segments, are perfused (Sweat et al., 2012). In light of these findings, the results from our current study suggest that vascular islands originating from network regression are capable of reincorporating into restimulated networks and we speculate that this process involves the re-endothelization of the collagen IV tracks.

The rat mesentery and compound 48/80 stimulation model were selected for this study to be consistent with our previous characterization of vascular islands (Kelly-Goss et al., 2012). The rat mesentery is a tissue that is actively remodeling, causing even unstimulated tissues to contain blind ended capillary vessels, as seen in Figure 2A. Evidence that at least a population of these blind ended vessels is associated with network growth is supported by adult rats having larger vascular areas than young rats (Hansen-Smith et al., 1994). Still, presumably their presence can be attributed to either vessel growth or regression. The latter case might explain the observation of vascular islands in the unstimulated tissues. We previously demonstrated that vascular islands were disconnected based on the comparison of PECAM labeling with injected fixable dextran labeling (Kelly-Goss et al., 2012). In unstimulated tissues, 40 kDa dextran injected via the femoral vein identified vessels throughout adult rat mesenteric microvascular networks, including the lumens of blind ended capillary segments, but not the disconnected endothelial cell segments associated with vascular islands (Kelly-Goss et al., 2012). In our current study, disconnected segments in day 70 post stimulation tissues were confirmed by confocal optical sections (Figure 4). An advantage of the rat mesentery is that it allows for observation of cells at different locations across the hierarchy of intact networks and enables analysis of vascular network area and length density (Figure 2), the presence of vascular islands (Figure 3) and cellular proliferation (Figure 8).

Rat mesenteric tissues can contain blood vessels only, lymphatic vessels only, both blood and lymphatic vessels, or no vessels at all. Our criterion for using a mesenteric tissue in the current study was that it had blood vessels. Thus, only a subset of the tissues used for our study contained lymphatic vessels (Figure 2C). PECAM-positive lymphatic vessels were distinguishable from blood vessels based on (1) a decreased labeling intensity, (2) larger relative diameters, and (3) more-uniform diameters across the hierarchy of a branching network. Also, at higher magnifications the PECAM labeling is more discontinuous along lymphatic vs. blood endothelial cell junctions (Baluk et al., 2007; Benest et al., 2008; Robichaux et al., 2010; Sweat et al., 2012). The lymphatic vessels in the rat mesentery identified based on the above PECAM labeling characteristics also label for typical lymphatic markers, including LYVE-1, podoplanin, and Prox1 (Benest et al., 2008; Robichaux et al., 2010; Sweat et al., 2012). Interestingly, vascular regression in this study seemed to be specific to blood vascular networks. In those tissues that contained lymphatics at day 70 post compound 48/80 stimulation, lymphatic vessels were commonly observed to still cover the entire tissue space (Figure 2C). Our laboratory's previous characterization of lymphangiogenesis and angiogenesis in response to 48/80 indicated that both processes occur by day 30, yet at this time point, blood vascular networks began to regress, while lymphatic vessel density remained the same (Sweat et al., 2012). The qualitative observations from our current study suggest that lymphatic vessels continue to occupy the entire tissue space at day 70 (Figure 2C). The idea that lymphatic vessels persist longer than blood vessels is consistent with previous work by Baluk et al. that characterized lymphatic growth in chronic airway inflammation (Baluk et al., 2007).

In summary, our results suggest that vascular islands originate during microvascular network regression and can become a source of endothelial cells for network regrowth. Collagen IV and pericytes along tracks left behind during regression implicate their role in re-connection of vascular islands to the nearby networks. While future work is required to understand the molecular mechanisms that regulate this process and its contribution to angiogenesis, this study adds to our fundamental understanding regarding endothelial cell dynamics involved in microvascular network growth.

### Conflict of interest statement

The authors declare that the research was conducted in the absence of any commercial or financial relationships that could be construed as a potential conflict of interest.

## Abbreviations

PECAM: platelet endothelial cell adhesion molecule
PBS: phosphate buffered saline
BrdU: bromodeoxyuridine
BSA: bovine serum albumin
LYVE-1: lymphatic vessel endothelial receptor 1.
